# Nitrous Oxide and Other Anæsthetics

**Published:** 1866-04

**Authors:** J. M. Carnochan

**Affiliations:** Surgeon-in-Chief to the State Emigrant’s Hospital, New York, etc., etc.


### SELECTIONS.

---

*Nitrous Oxide and other Anæsthetics.*---By J. M. CARNOCHAN, M. D., Surgeon-in-Chief to the State Emigrant's Hospital, New York, etc., etc.

I desire to present through the pages of the "Medical and Surgical Reporter," a general statement of the facts respecting three surgical operations which I performed, using nitrous oxide gas, administered by Dr. Colton, as the anæsthetic, and my opinion on the value of this agent as compared with chloroform and ether.

The first operation took place on the twenty-second of last July, and was the removal of the entire breast, and glands of the axilla, for cancer. The patient, a lady in feeble health, was suffering from disease of the throat and lungs and general debility. In thirty-five seconds from the time she began inhaling the gas, she was in a profound anæsthetic sleep. She remained insensible for sixteen consecutive minutes, until the operation was completed, and in forty seconds, from the time the bag was removed, awoke to consciousness without nausea, sickness, or vomiting, as is so often the case with inhalation of chloroform and sulphuric ether.

The second and third capital operations occurred at the State Emigrant's Hospital, on the second of December, and consisted of two amputations of the leg. The time required to produce an anæsthetic sleep in the first patient, a male adult, extremely debilitated and worn out by disease, was forty-five seconds; whole duration of the operation and influence, two minutes and a quarter. No nausea or unpleasant symptoms.

The third operation was on a boy of about thirteen years of age. The time consumed in the inhalation, operation, and recovery from the anæsthetic sleep was two minutes, the gas working equally as in the other cases, and the patient, after complete anæsthesia, awaking entirely free from unpleasant symptoms.

For minor operations, or for capital operations, such as amputations, which, when properly performed, should require but a few minutes, I have no hesitation in stating that the nitrous oxide gas, as an anæsthetic, is far superior to either chloroform or ether. Insensibility is suddenly produced, and the patient recovers consciousness quickly, the operation being attended by no nausea or sickness, and without the dangerous effects often incident to chloroform and ether.

It is worthy of remark that the nitrous oxide gas approximates, in its chemical combination, to the composition of the ordinary atmosphere, and we may thus, inferentially, account for its more favorable influence. Whether it can be used in operations, which from their nature require from half an hour to an hour's time, remains still to be proved by actual experiment.

The duration of the anæsthetic influence in the case of the first operation, previously alluded to, is the longest on record; and I may here state that this is the first capital operation performed under the influence of the gas, since the great discovery of Wells, of Hartford, twenty-two years ago, that a harmless sleep could be produced by a chemical agent, which could annul for the time being the greatest suffering. It is not at all improbable that had Wells lived and had the boldness to follow up his early successful experiments, chloroform and ether would never have been thought of as anæsthetics.

To T. G. Colton is due the credit of reviving the use of this important agent, in the practice of dentistry, after a lull of twenty-two years.

The value of a safe anæsthetic agent, which can be used without anticipation of danger by the patient, is a great boon to suffering humanity, and I have related thus minutely its action in my own cases, in the belief that similar favorable results met with by others in the nitrous oxide gas will supersede all other anæsthetics now in use.---*Med. and Surg. Reporter.*

